# Developing Adaptive Serious Games for Children With Specific Learning Difficulties: A Two-phase Usability and Technology Acceptance Study

**DOI:** 10.2196/25997

**Published:** 2021-05-31

**Authors:** Oguzcan Yildirim, Elif Surer

**Affiliations:** 1 Department of Modeling and Simulation Graduate School of Informatics Middle East Technical University Ankara Turkey

**Keywords:** serious games, adaptive games, specific learning difficulty, usability, system usability scale, technology acceptance model, training, development, adaptation, gaming, learning disability, children, education, teacher

## Abstract

**Background:**

Specific learning difficulties (SpLD) include several disorders such as dyslexia, dyscalculia, and dysgraphia, and the children with these SpLD receive special education. However, the studies and the educational material so far focus mainly on one specific disorder.

**Objective:**

This study’s primary goal is to develop comprehensive training material for different types of SpLD, with five serious games addressing different aspects of the SpLD. The second focus is measuring the impact of adaptive difficulty level adjustment in the children’s and their educators’ usability and technology acceptance perception. Receiving feedback from the children and their educators, and refining the games according to their suggestions have also been essential in this two-phase study.

**Methods:**

A total of 10 SpLD educators and 23 children with different types of SpLD tested the prototypes of the five serious games (ie, Word game, Memory game, Category game, Space game, and Math game), gave detailed feedback, answered the System Usability Scale and Technology Acceptance Model (TAM) questionnaires, and applied think-aloud protocols during game play.

**Results:**

The games’ standard and adaptive versions were analyzed in terms of average playtime and the number of false answers. Detailed analyses of the interviews, with word clouds and player performances, were also provided. The TAM questionnaires’ average and mean values and box plots of each data acquisition session for the children and the educators were also reported via System Usability Scale and TAM questionnaires. The TAM results of the educators had an average of 8.41 (SD 0.87) out of 10 in the first interview and an average of 8.71 (SD 0.64) out of 10 in the second interview. The children had an average of 9.07 (SD 0.56) out of 10 in the first interview.

**Conclusions:**

Both the educators and the children with SpLD enjoyed playing the games, gave positive feedback, and suggested new ways for improvement. The results showed that these games provide thorough training material for different types of SpLD with personalized and tailored difficulty systems. The final version of the proposed games will become a part of the special education centers’ supplementary curriculum and training materials, making new enhancements and improvements possible in the future.

## Introduction

Special education is a form of education that is customized to the needs of children who have a disability, illness, or difficulty. In the United States, special education is offered in public schools, and this right is secured by the Individuals with Disabilities Education Act (IDEA) [1]. IDEA categorizes disabilities and conditions demanding special education into 13 types, including autism, attention-deficit/hyperactivity disorder, and specific learning difficulty (SpLD). A specific learning impairment (also known as a specific learning disability or SpLD) is a condition in which children learn specific concepts at a slower rate than would be expected for their age or educational level. Learning disability (or learning disorder; LD) is not associated with SpLD, although it is a medical term used for diagnosis and is often referred to as LD. To avoid any misunderstanding, the word SpLD will be used in this study to refer to specific learning difficulties. SpLD is not linked to intelligence, which means that children with SpLD may have average or superior intelligence but learn slower than their peers. SpLD is divided into five groups by the Learning Disabilities Association of America as dyslexia (reading disability), dyscalculia (math disability), dysgraphia (writing disability), oral and written language disorder and specific reading comprehension deficit, and nonverbal learning disability.

Dyslexia is the most well-known form of SpLD, and it causes spelling mistakes, poor reading, and false reading in children. Children with dyscalculia have difficulties with numbers and abstract principles such as counting and basic numerical operations. Misspelling, writing letters in wrong forms, and writing letters of various sizes in a word are all symptoms of dysgraphia in children. Oral and written language disorder and specific reading comprehension deficit affect children’s ability to articulate and comprehend sentences. Dyspraxia can show itself in various behaviors, from a lack of coordination between the lips and tongue to problems with handwriting. There is no agreement on the types of SpLD, and the coverage of SpLD changes in every country. Since the proposed games in this study are intended for the Turkish training system and given that these SpLD types are accepted as SpLD in Turkey [2] and are suggested by educators in special education centers with whom the authors collaborated, they are accepted as SpLD types in this paper. In Turkey, 41,600 people have been diagnosed with SpLD, and 82% of dyslexic students drop out of school before attending university. To date, the only verified therapy for SpLD is special education. In addition to the regular school education, children with SpLD attend extra classes taught by special education teachers in groups or individually. This special education can include reading, writing, algebra, developing motor coordination skills, and language education activities.

The impact of games on children’s training with SpLD has been discovered recently, and the number of studies on this topic has increased. Investigating the influence of daily video game playing on children with SpLD was the initial research focus in SpLD research [3]. In a study [4], researchers collaborated with 20 children with dyslexia to see if video games, Rayman Raving Rabbids, a promotional Wii video game, influenced their reading abilities. In another study [5], an app, which included six mini-games, was created to diagnose dyslexia in children aged 6 to 7 years. Each of the games in the app tested a different ability such as word forming, syllabic memory, verbal work memory, auditory memory, and word reading. Teachers found the app helpful in identifying children with potential dyslexia and correcting exercises. Nonetheless, the app was language-dependent, and children should have been able to read and write. Another app [6] for screening dyslexia in children aged 7 to 12 years was also developed to solve the language dependency. There are also findings in the SpLD literature for dyscalculia. A serious virtual reality game was created to help children with dyscalculia learn math [7]. Three separate scenarios and game plays were included in the game, each with three different difficulty levels. The participants in the study were 40 students aged 7 to 9 years. The suggested game was played by half of the children at random, while the other half, the control group, was handled with the conventional Domino system. Children who played the proposed game took substantially less time to fit simple mathematical operations to the correct answer when compared with the control group.

In a study performed in Germany [8], a mobile game–based intervention on syllable stress and literacy was developed for German children with dyslexia. The study focused mainly on the design criteria of the game-based intervention, while the quantitative analysis was planned to be performed on children by using the mobile game 20 minutes a day, 5 days per week. Similarly, Sood et al [9] also focused on developing games where the objectives were detecting, monitoring, and managing dyslexia in young children (aged 4-18 years). The study presented the details of the protocols and game design principles, but the games were not tested with the control group yet. Flogie et al [10] developed intelligent game interventions and used them with 51 children with learning difficulties in the mainstream Slovenian education system. The results showed that intelligent game interventions provide personalized education and can be helpful while designing the specialized curriculum.

This study is adapted from author OY’s Master’s thesis [11], supervised by author ES. This study concentrates on serious games’ usability and technology acceptance results in the training of children with SpLD. As previously mentioned, serious games, or games with more than just entertainment value [12,13], have recently become an important research topic among researchers interested in children’s education with SpLD. To the best of the authors’ knowledge, this is the first systematic research covering all types of SpLD and measuring the impact of using adaptive difficulty via quantitative and qualitative approaches. This research’s primary aim is to create a collection of specially developed games for use in special education for children with any form of SpLD. Usability tests were used to investigate the potential of this series of games. Another aim is to demonstrate how the adaptive difficulty system affects children’s playing experience. An adaptive difficulty framework was developed to assess its impact on children with SpLD. To that end, five serious games for children with SpLD were designed and developed. Two of the five games were improved with an adaptive difficulty system to increase the children’s experience when playing the games. Children with SpLD and their educators evaluated all five games, and their responses to questionnaires—System Usability Scale (SUS) [14] and Technology Acceptance Model (TAM) [15]—and their comments on the games were classified and analyzed. The results demonstrated the games’ usability as enhanced training material in special education. Both the children with SpLD and their educators gave highly positive feedback regarding playing the established games, which was also repeated on the responses to the SUS and TAM questionnaires. This study was split into two parts to implement the children’s and educators’ recommendations into the framework and thoroughly analyze the outcomes.

## Methods

### Overview

For this study, five different serious games were designed and developed to train children with any type of SpLD. The details of the games and their versions are explained in detail in the following subsections. The games were developed in Unity (version 2018.3.0f2), a cross-platform game engine that allows its users to build games in 2D and 3D environments. For this study, an ASUS Zenpad 8 tablet is used during the data acquisition so that Unity’s Android device settings were used during the game development procedure. After the game development process, the games were tested twice by two participant groups, including 10 educators and 23 students with SpLD between the ages of 7 and 11 years. During the first interview with the participant groups, the SUS and TAM questionnaires were filled by the participants besides applying the think-aloud methods during game play and answering open-ended questions after playing the games. After the first interviews, a rule-based adaptive difficulty enhancement was added to two of the five games to measure the impact of adaptiveness in the developed serious games. During the second interview with the children, only open-ended questions and think-aloud methods were used. For the educators, the same interview procedure was used as before. Multimedia Appendix 1 summarizes the prototyping and interview procedures of the study.

### Design Criteria and Themes of the Games

At first, with special educators’ help, design criteria were defined to develop games compatible with the children’s educational background. The first criterion is about the games’ target groups, including children with SpLD who know reading and writing. Therefore, children’s age was limited to 11 years since designing a decent game for both new readers and secondary school students could be an unfeasible goal. The second criterion is that games should be easy-to-use given the children’s struggle with SpLD. Thus, games do not include multitasking such as picking up stars in the sky while solving multiplication problems on the ground.

Given that children with SpLD will play the games, the themes of the games were decided cautiously. Games do not focus on a single type of SpLD but cover various types of SpLD. The first game’s theme was the spelling for children with dyslexia or dysgraphia. Simple mathematical operations were the second game’s theme for children with dyscalculia. The third game’s theme was writing examples of specific categories developed for children with dysgraphia or dyslexia. Two more themes were added for different purposes (ie, a memory game targeted at improving visual memory and a space game requiring time management and constant concentration). When choosing themes, the educators showed what kind of practices they do in their special education and suggested game ideas, so the opinions of educators were also taken into consideration during the game design. The children had only 30 minutes per week, and they had to play all the games and answer the study questions in 2 weeks, so the number of themes was limited due to the time limitations of the children.

### Developed Games

During this study, different versions of the five games were developed. The first version of the games were developed as prototypes, but all the themes and rules were already well-defined. Educators examined the games’ prototype versions so that the educators’ first interview could be considered an initial quality check (Prototype I). Since the users requested no modifications after Prototype III, only two prototypes were developed in the Word game. The Category game and Math game had three prototypes in total for the same reason (ie, no updates were requested by the users). Four different prototypes were developed for the Memory game and Space game.

### Word Game: Prototype I

**Figure 1:**
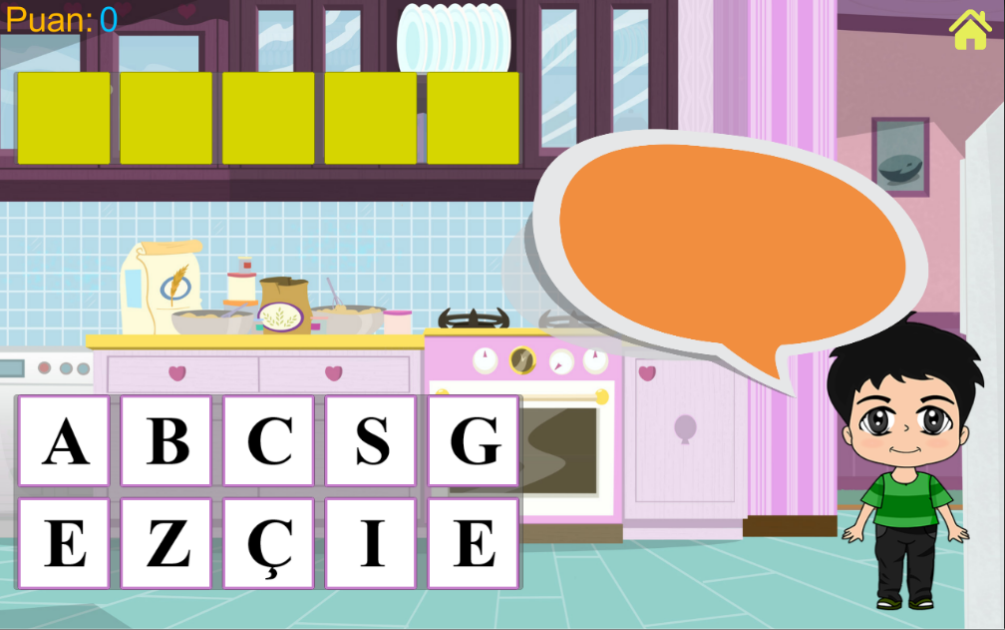
Screenshot of the Word game from Prototype I.

The word game (Figure 1) is focusing on spelling for children with dyslexia or dysgraphia. In the game, a child character asks 10 questions about a single predefined topic, and the answers are limited to 4 to 6 letters in Turkish. The player needs to drag and drop the letters to the appropriate yellow squares, representing a letter in the answer. If the letter is correct, it replaces the yellow square, and if it is a false letter, the letter does not fill the yellow square and returns to its initial position (bottom left corner). After entering all the correct letters, the child avatar confirms “It is correct!” and asks the next question. The game’s background image is chosen to keep the players focused on the topic. For instance, “Kitchen” was the topic of the first designed level, and the players were asked to answer questions about cutlery or fruits, such as “What is the name of the sweet fruit which has red, yellow, and green colors?” and “What do you use to eat your soup?” The player gets 10 points for each correct answer.

### Word Game: Prototype II

A skip button was added to the screen so that unsolved questions could be passed. The number of correct answers, the number of false letters, the number of skipped questions, and the total time were recorded in the database.

### Word Game: Prototype III

Given that the users did not request any new updates after Prototype II, this game was not modified.

### Word Game: Prototype IV

Given that the users did not request any new updates after Prototype II, this game was not modified.

### Memory Game: Prototype I

**Figure 2:**
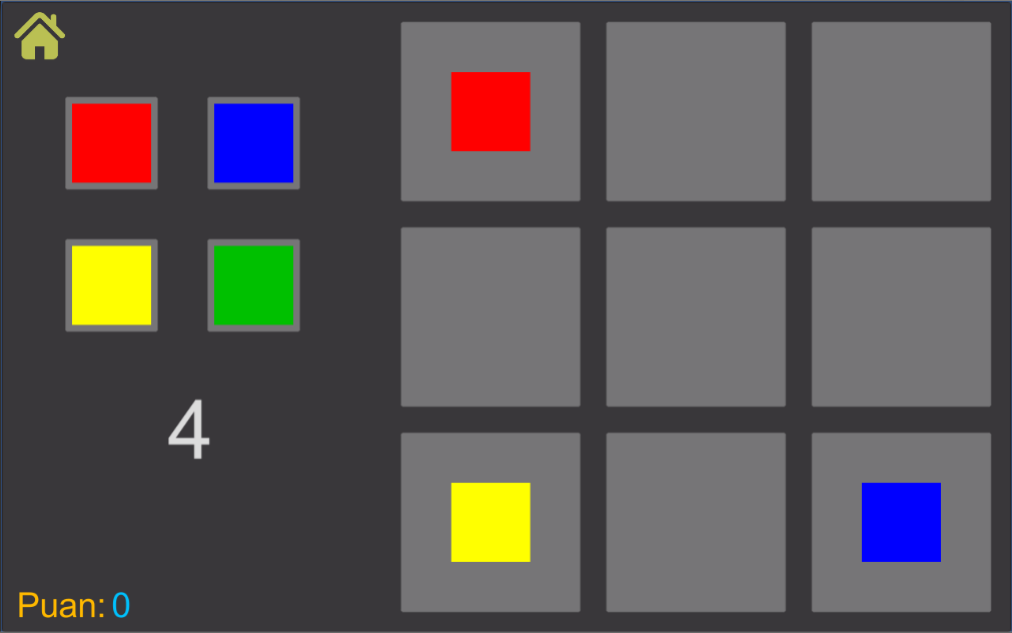
Screenshot of the Memory game from Prototype I.

The Memory game (Figure 2) was created to improve the visual memory and attention of children with SpLD. In the game, there are two panels; on the left side of the screen, there are four colors (or four arrows each faced in another direction) and a counter. Nine boxes are aligned as a three-by-three grid on the right side of the screen. When the game starts, initial colors appear on three of the nine boxes randomly, and the counter starts to go down. After the counter hits zero, from five, colors on the right side disappear. The player’s goal is to refill the three boxes with the correct colors as quickly as possible. The player first needs to tap the correct color on the left panel and tap the right panel’s accurate grid. To erase the grids, the player can tap on a filled grid. Whenever the player fills all three grids correctly, “CORRECT!” feedback shows up at the counter’s position. After 3 seconds, a new randomly generated memory question is asked, where each correct answer is 10 points.

### Memory Game: Prototype II

A hint button was added to the screen to show the colors or arrows once again to the players. Additionally, a timer was added to display the countdown. Both the difficulty and timer can be set just before starting the game by the educators or parents. The difficulty setting affects the number of grids to be remembered during the game. The number of correct answers, number of chosen false grids, number of grids filled with wrong color or direction, difficulty level, and session time were recorded in the Memory game’s database.

### Memory Game: Prototype III

After analyzing the first two prototypes, two games—the Memory game and Space game—were updated and enhanced with the adaptive difficulty level. The games’ design or mechanics were not modified. The purpose of the second interview with the students was to determine their behavior and success in games with adaptive difficulty levels.

### Memory Game: Prototype IV

To represent the alternative versions of this game, a version with pictures was developed (Multimedia Appendix 2). In this version, sky objects were used, such as sun, moon, cloud, and lightning. Moreover, the appearing time can be set as 3, 4, or 5 seconds before the game starts, increasing the difficulty. Thus, this game has four versions (colors, arrows, letters, and pictures), five difficulty options—one grid, two grids, three grids, four grids, and adaptive (two grids with variable appearing time)—with three different appearing time options (3, 4, and 5 seconds), and an adjustable timer.

### Category Game: Prototype I

**Figure 3:**
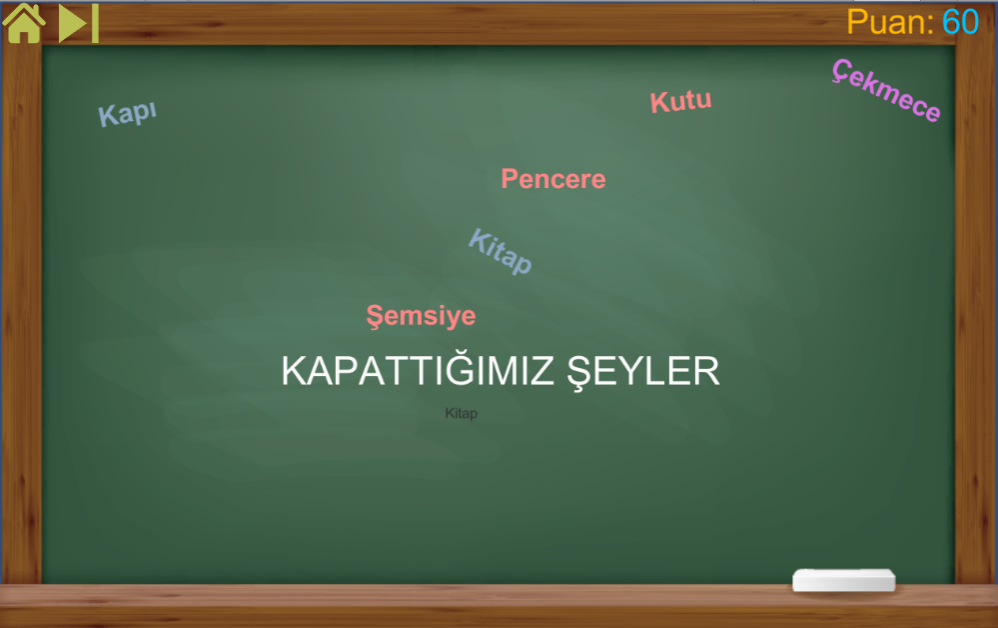
Screenshot of the Category game from Prototype I.

The Category game was developed to improve the thinking and writing skills of children with dysgraphia or dyslexia—not handwriting but correctly ordering letters in a word. During the game, a couple of categories are given, and the players are expected to find words related to that category. For example, in Figure 3, the category is “Things that we close,” and predefined words are “Window,” “Door,” and “Box.” In each category, there are 15 to 20 predefined answers. If the player writes down one of them, it appears on the screen with a random color and position, and the player gains 10 points. If the answer is wrong or misspelled, it does not appear on the screen. The player uses the keyboard of the tablet to write the words. If the player gets stuck at some point, the skip button on the top right corner can be used, and a new category starts. There were 10 different categories in the first version of the game.

### Category Game: Prototype II

The category game was separated into two parts. In the first part (Multimedia Appendix 3), the player must choose five correct words about a given category, between 20 available options. After the fifth correct answer, the category changes. When five categories are completed, the second part, which is the same as the previous Category games, starts. The player gives examples of the same categories in the first part. Moreover, a skip button is added to the screen. Session time and the number of correct and false answers were saved in the database. For the second part (Multimedia Appendix 4), the number of correct answers, number of typos, number of false words, and session time were recorded.

### Category Game: Prototype III

In this prototype (Multimedia Appendix 5), the input system of the open-ended questions was modified. The player does not use the keyboard of the tablet keyboard anymore but uses an on-screen keyboard aligned in alphabetical order.

### Category Game: Prototype IV

Given that the users did not request any new updates after Prototype III, this game was not modified.

### Space Game: Prototype I

**Figure 4:**
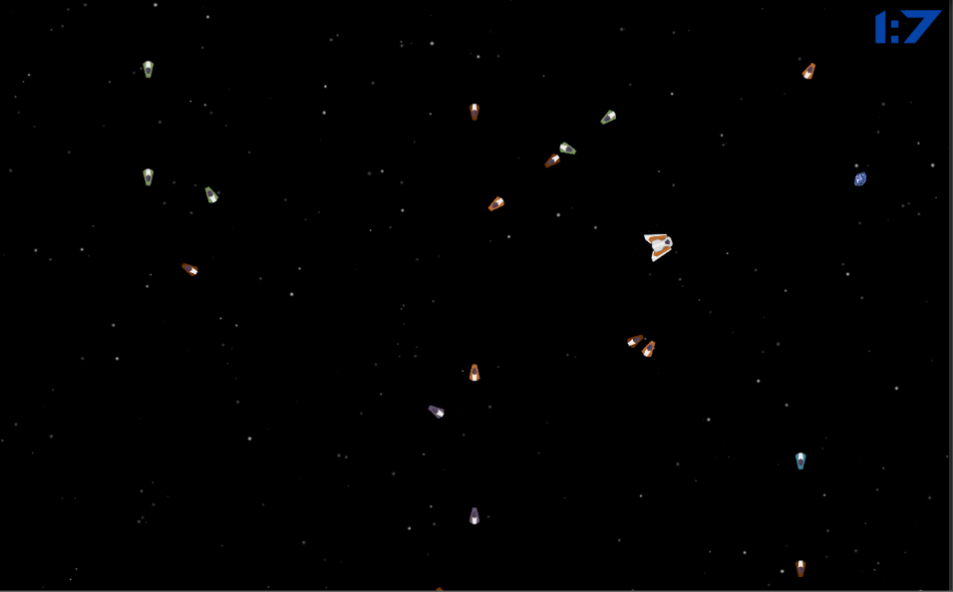
Screenshot of the Space game from Prototype I.

In the Space game (Figure 4), the primary objective is to test the time management and concentration levels of children with a theme that is not causally related to their courses. The player’s primary goal is collecting gems while trying not to hit tiny spaceships that are programmed to go in a random direction. The initially available time slot is 90 seconds, and the timer counts down. Whenever the player taps into the screen, a spaceship rotates into that point and moves toward the point until it reaches the tapped position. A gem appears every 5 seconds at a random location. If the player cannot collect the gem in 10 seconds, the gem disappears. Every 0.4 seconds, an enemy spaceship spawns off-screen and flies straight through the screen in a random direction. The player gains 2, 4, or 6 seconds randomly after collecting a gem. Whenever a player collides with another spaceship, the player loses 3, 5, or 7 seconds randomly. The game is over when the time is up.

### Space Game: Prototype II

No modification was applied in the Space game except forming a database for game statistics. The number of collected gems, the number of crashes with the enemy, and session time were adjusted to be kept in the database.

### Space Game: Prototype III

After analyzing the first two prototypes, two games—Memory game and Space game— were updated and enhanced with the adaptive difficulty level. The games’ design or mechanics were not modified. The purpose of the second interview with the students was to determine their behavior and success in games with adaptive difficulty levels.

### Space Game: Prototype IV

All the objects in the scene, except the user interface, were enlarged. The control mechanism was changed with a joystick, which was added to the screen’s bottom right. Additionally, the default enemy spawn frequency was decreased. The waiting time between two enemies’ spawn was changed to 0.7 seconds. Moreover, visual feedback on collecting gems and crashing into enemies was added. Players could also see how much time they gained (in blue) or lost (in red) during the game.

### Math Game: Prototype I

**Figure 5:**
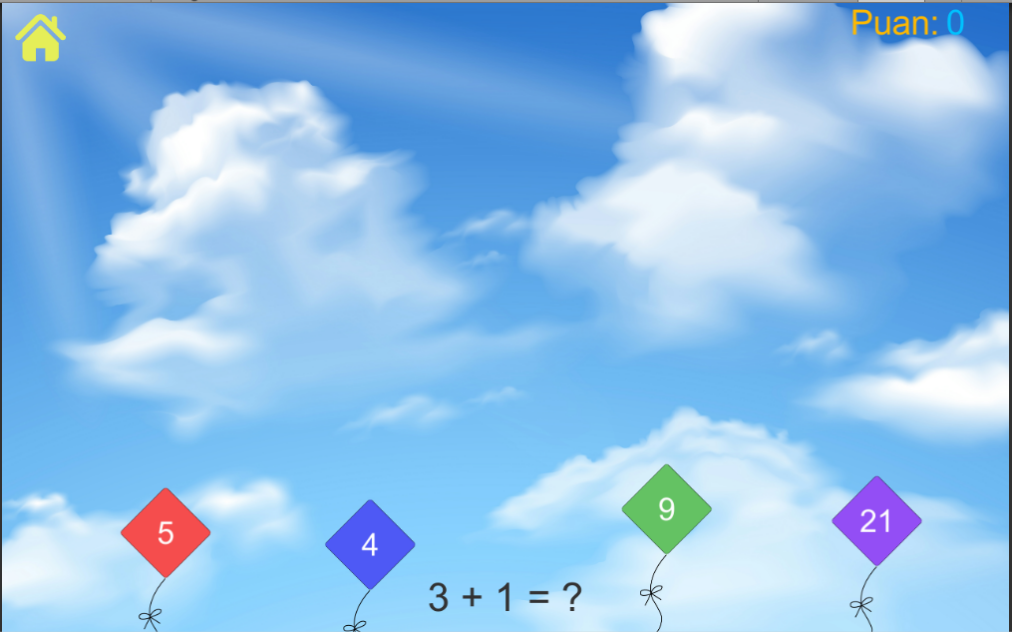
Screenshot of the Math game from Prototype I.

The Math game (Figure 5) is a simple addition and subtraction game. In the first version, the math question appeared below the screen, and four kites floated up in the air. Questions are created randomly with a set of selected numbers between 0 to 15. The player’s primary goal is tapping on the kite containing the correct answer before exiting from the screen. There was no penalty for choosing wrong answers to see the tapping strategy of the children. They could tap all four to see the next question and still get total points.

### Math Game: Prototype II

A timer was added as a countdown, and both the timer and difficulty could be set before the game starts. The difficulty level modified the kites’ speed, and a maximum limit of numbers for the math operations could also be arranged. The number of correct and false solutions, unanswered questions, session time, and difficulty level were kept in the database.

### Math Game: Prototype III

As an option, the multiplication operation was added to the game, and the scoring system was modified so that the player does not gain total points after their first guess. After each guess, the scoring system changes (ie, in the second guess, 8 points; in the third guess, 4 points; and a the last guess, only 1 point is given to the player).

### Math Game: Prototype IV

Given that the users did not request any new updates after Prototype III, this game was not modified.

### Adaptive Difficulty Mechanism

The adaptive difficulty is designed to check consecutive successful and unsuccessful moves by the player and adjust the game difficulty according to the player’s game play level. A simple adaptive difficulty system was developed for the Memory game and Space game. The number of games was decreased to two since these games would be played by the students twice, and the students had time constraints. Besides, these two games had repeatability, and they were liked by children the most. This adaptive difficulty was added to these games to keep the players in the *golden path* or *optimal game play corridor*, which prevents the players from getting bored or frustrated by the difficulty of the game, as it is introduced in the Flow study of Czikszentmihalyi [16] and enhanced by Thomas and Young [17] (Multimedia Appendix 6). For the adaptive difficulty, a rule-based method was selected due to the time constraints of the students. They could only play both games twice, once each for the standard and adaptive versions. Since repetitive testing is no longer an option, methods that use training could not be used. The rule-based difficulty method was chosen to modify the difficulty for a couple of actions instead of every action. These two alternatives would give the same results for large sample sizes in theory; however, the rule-based method was chosen to ensure that every student gets the same results for their successful or unsuccessful set of actions. In addition, since there will be only one game play session per student, the adaptive difficulty should affect the game while the game is continuing, unlike the study of Hendrix et al [18] where the players’ previous game sessions changed the game difficulty.

The system was designed to change only one variable to get meaningful results from the adaptive difficulty levels. For the Memory game, the correct answer’s display time was the selected variable, while for the Space game, enemy spawn frequency was selected. These variables affect games directly, but they cannot be noticed by the player explicitly, unlike the number of letters in the Memory game or the type of enemies in the Space game, so that the students can give their honest opinion about how they feel about the game difficulty without any a priori clue. The flow of adaptive difficulty change is shown in Multimedia Appendix 7. Since the students could play these games only for a limited time, a single change in the variables was adjusted by around 10%. The difference in these variables could affect the outcome quickly after the game gets easier or more difficult.

### Memory Game With Adaptive Difficulty System

**Figure 6:**
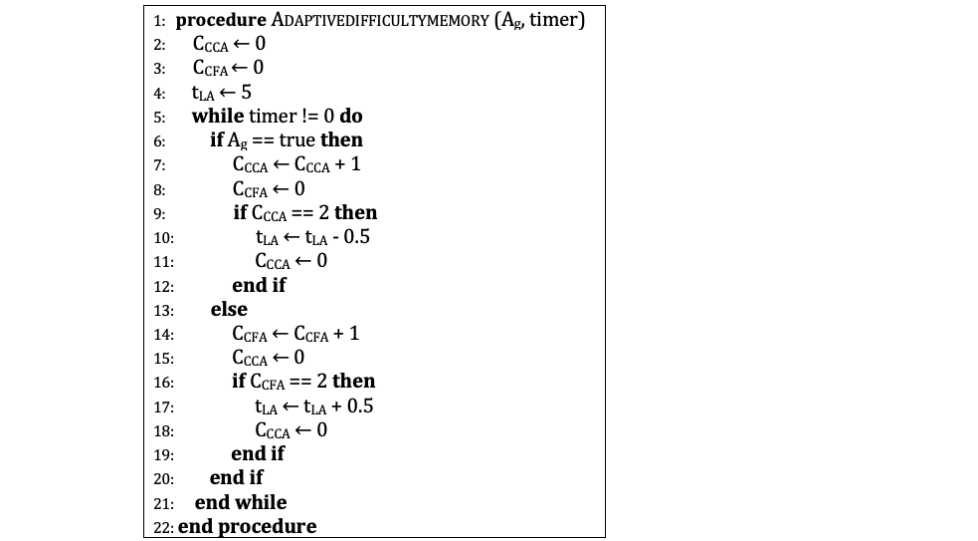
Adaptive difficulty memory.

For all the students with dyslexia or dysgraphia, a letter version of the Memory game was developed. The Memory game’s letter version (Multimedia Appendix 8) contains only four consonants: *p*, *q*, *b*, and *d*. These are the most commonly confused letters for children with dyslexia when reading and writing. Although the Turkish alphabet does not contain the letter *q*, children are familiar with this letter from the keyboard. The adaptive system modifies the first appearing time of the letters on the screen, and the timer counts down seemingly from five. However, it counts faster or slower according to the success of the players in the game. The rules of the adaptive difficulty are simple, as they are explained in Figure 6 in pseudocode. If the player can give two correct answers (C_CCA_) in a row, the appearing time (t_LA_) decreases by half a second. If the player provides more than one false solution, false grid, or incorrect letter in a row (C_CFA_ is used for all), the appearing time increases by half a second (A_g_ is used for *given answer*). Changes in the emerging time are applied to the next question.

The first interview results show that students gave an average of 4.45 correct answers and 2.35 false answers for two grid difficulties in 60 seconds, which led to the selection of two consecutive correct or false answers as the adaptive difficulty changes condition. According to the results, the expected average difficulty change was more than two for the increment and more than one for the decrement during 100 seconds of playtime. Due to these initial outcomes, the amount of the change was set as 0.5 seconds so that neither students can realize the difference directly nor face substantial difficulty changes. The number of correct and false answers before a change in appearing time and the number of changes in appearing time were added to the database.

### Space Game With Adaptive Difficulty System

**Figure 7:**
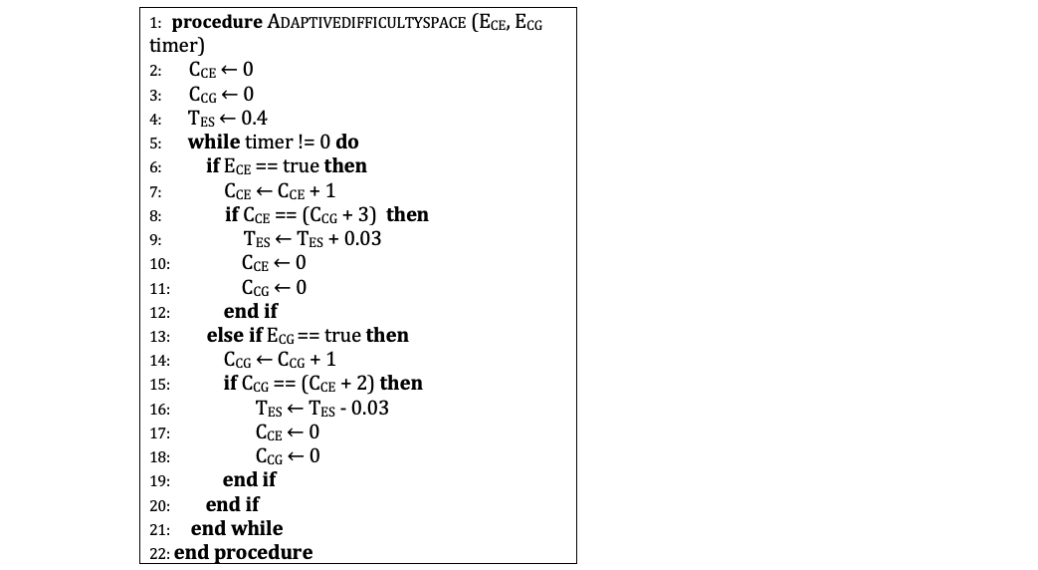
Adaptive difficulty space.

Adaptive difficulty in the Space game only affects the enemy spaceships’ spawn frequency (Figure 7). If the player is successful, the frequency increases, and vice versa. The default waiting time (T_ES_) between the spawn of two enemy spaceships is 0.4 seconds. The waiting time increases by 0.03 seconds when the number of crashes (C_CE_) is three more than the number of collected gems (C_CG_).

Waiting time decreases by 0.03 seconds when the number of collected gems is two more than the number of crashes. Both counters reset when the spawning frequency changes (E_CE_ is used for the crashing enemy event, and E_CG_ is used for the collect gem event). According to the first interview results, students collected gems an average of 0.997 times. They crashed the enemies an average of 1.317 times per 10 seconds, and their average playtime was 94.06 seconds. Due to these results, the number of events (crashing enemies or collecting gems) is stored as one variable. Instead of the consecutive events, to change the difficulty, the total number of crashes or collected gems is expected to be more than the other. To decrease the enemy spawn period, the number of collisions is expected to be three more than collected gems instead of two since the students were more likely to crash into an enemy rather than collect a gem. The change amount of the enemy spawn period was selected as 0.03 seconds since the Space game is a more dynamic game that requires more attention than the Memory game. The change was kept below 10% to avoid a profound difference.

### Participants

In this study, 10 educators participated in the study—7 of them were special education teachers, 2 of them were occupational therapists, and 1 was a psychologist. The mean age of educators was 33 (SD 14.37) years, and 7 of the educators were female. Children participants were selected among the Albatros Special Education Center (Albatros Özel Eğitim Merkezi in Turkish), which provides education for children with SpLD. The final student group includes 23 students in the age group of 7 to 11 years. Before the data acquisition started, permission of their parents was taken both verbally and written. Additionally, children’s consent was taken verbally before the acquisitions, and the voluntary nature of the study was emphasized. Only 3 children could not complete the study since they had changed their school before the end of the study. The mean age of the 23 children participants was 8.6 (SD 1.13) years. Most of the children were male (18/23, 80%). A total of 20 children were diagnosed with the SpLD by a doctor. The rest of the participant children had taken the Albatros Special Education Center’s test. Given that the special education center’s psychologists evaluated them as having SpLD, they were also included in the study.

### Permissions and Ethics Approval

This study was performed by two participant groups—students and their educators. This study was not designed as a clinical trial but as a two-phase usability study for the Master’s thesis of OY, supervised by ES. The Ethical Approval of Research was approved by the Middle East Technical University Human Subjects Ethics Committee (ID of the permission: 28620816/398). The participants or their parents, if they were children, were informed both with verbal and written communication about the purpose of the study, possible benefits of the study to children with SpLD, and the voluntary nature of participation.

### Data Analysis

For both participant groups, the box plot of SUS and TAM questionnaires were plotted using SPSS Statistics 25 software (IBM Corp), while the individual plots were plotted with the Minitab software’s trial version (Minitab, LLC). For each interview and questionnaire, the average and SD of the positive statements for each game type were calculated. Student’s game statistics, including the average play time, the number of correct answers, and difficulty-based changes, were also presented in detail. In addition, the Wilcoxon signed rank tests for both SUS and TAM questionnaires were calculated. This test was applied to compare the standard and adaptive versions of the games since data were not normally distributed.

## Results

### Overview

In this section, data acquired from the interviews are analyzed. Throughout this study, two interviews with both participant groups (students and educators) were done. Answers to the following questions were examined:

- Are proposed games suitable for training purposes?
- Are children with SpLD comfortable with different types of the proposed games?
- Can an adaptive difficulty system provide a better game experience for children with SpLD?

### The Questionnaire Results of the Students

There are 10 questions in the SUS, where half of the questions consist of positive statements. The TAM questionnaire includes 19 questions about games to understand participants’ acceptance level of the proposed games (Table S1 in Multimedia Appendix 9). The playtime of the games was recorded, and the average playing times are shown in Table S2 in Multimedia Appendix 9.

### The Comments and Suggestions of the Students

The comments of children (Multimedia Appendix 10) were examined in three sections: comments on current games, future game ideas, and general feedback. For the first part, children were asked to comment on existing game ideas, the difficulty level of games, visuals, and all related things to the games. Most children said that the Math game was too simple in terms of visualization and the question types. They all said that there should be other operations (multiplication and division), and there should be more themes such as planes and cars besides kite themes. Multiplication was added to the final version of the game. Most of the students complained about the number of enemies and their small sizes in the Space game, which were fixed in the final prototype. Most of the students wanted to play the Space game level by level rather than in an endless fashion. In addition, they said that they wanted to see “boss creatures” at the end of each level. Moreover, most children suggested alternative control mechanisms for the Space game, such as buttons, touch joystick, or dragging. After these suggestions, a touch joystick was added in the last version. There were not many common comments on the Word game. Finally, students were asked if they had any other comments in general. Three of them suggested that music or audial feedback in the game will make it more fun. Most of the children proposed an in-game achievement system. Finally, one of the children suggested a ranking system so that players can understand if they won something or not. Multimedia Appendix 11 shows the most common words or phrases in the comments of students. The most common five words were *colorful*, *ship*, *car*, *control*, and *enemy*.

### Game Statistics of the Students

In this section, statistics of the games are shown. Tables S3 and S4 in Multimedia Appendix 9 show the data of students in the Memory game from the first interview. *Number of false grids* refers to when the player fills a grid that is supposed to be empty. *Number of false color/arrow markings* refers to cases when the player marks a grid with a wrong color or arrow option. The difficulty of the Memory game changes with the number of grids since they should be memorized to be filled. Students played this game three times: the first color version with low difficulty to get used to the game, then again the color version with greater difficulty, and finally the arrow version. The level of difficulty was set according to the age of the students.

Tables S5-S7 in Multimedia Appendix 9 show the students’ game statistics from the first interview of the Space game, Math game, and Word game, respectively.

Statistics of both parts of the Category game are shown in Tables S8 and S9 in Multimedia Appendix 9. For part two, an irrelevant answer to the given category is counted as *False Answer*, while misspelled words are counted as *Typo*. Table S10 in Multimedia Appendix 9 shows the students’ data in the Memory game from the second interview, where they played each version of the game once. The Space game statistics from the second interview with the students are shown in Table S11 in Multimedia Appendix 9.

Table 1 shows how often the adaptive difficulty system adjusts the difficulty while the students play the games.

**Table 1:** Adaptive difficulty changes during game: mean and SD results of in-game adaptive difficulty changes for both games in the second session with students.

Game	Difficulty up, mean (SD)	Difficulty down, mean (SD)
Memory game	2.58 (1.41)	1.25 (0.97)
Space game	1.52 (1.20)	1.56 (0.98)

**Figure 8:**
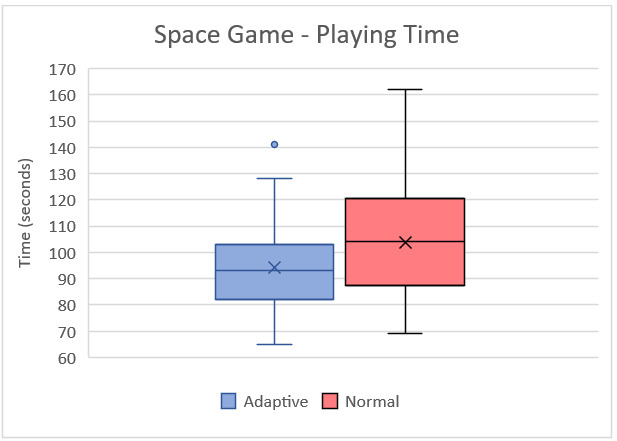
Box plot of playing time for the Space game from the second session with students.

**Figure 9:**
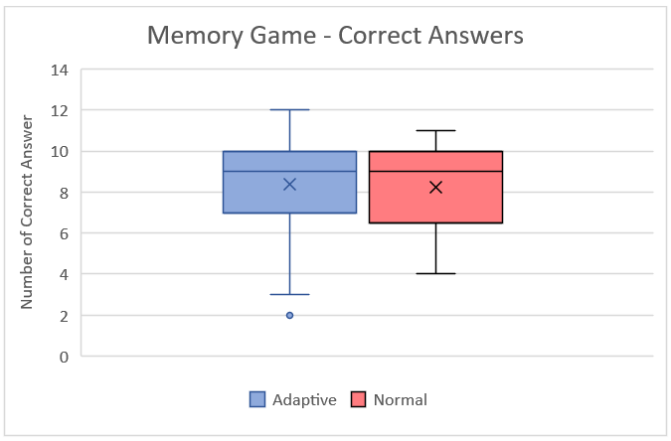
Box plot of correct answers for the Memory game from the second session with students.

Wilcoxon signed rank test results can be seen in Table 2. This test was applied to compare the games’ standard and adaptive versions since data were not normally distributed. Column *Z* refers to the *z* score, which is closer to 0 when the groups are evenly distributed. A P value of .05 is approximately equal to a *z* score of 1.96. The asymptotic significance column shows the significance of the difference. A value of less than 0.05 is considered significant. Figures 8 and 9 show the box plots of playing time for the Space game and Memory game, respectively.

**Table 2:** Wilcoxon signed rank test: results of test between data of two versions of both games from the second session with students.

Game	*Z* score	Asymptotic significance (two-tailed)
Memory game: correct answers	–0.567	0.571
Memory game: false grid	–0.138	0.890
Memory game: false letter	–0.536	0.592
Space game: crashed gem	–2.173	0.030
Space game: crashed enemy	–0.924	0.355
Space game: playtime	–1.884	0.060

### The Questionnaire Results of the Educators

**Figure 10:**
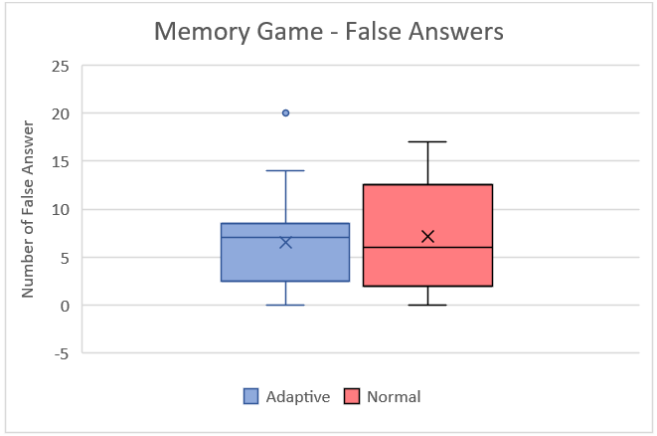
Box plot of false answers for the Memory game from the second session with students.

**Figure 11:**
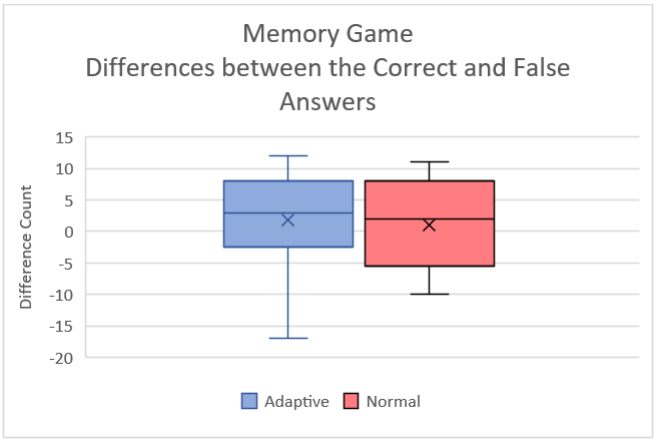
Box plot of the difference between the correct and false answers for the Memory game from the second session with students.

**Figure 12:**
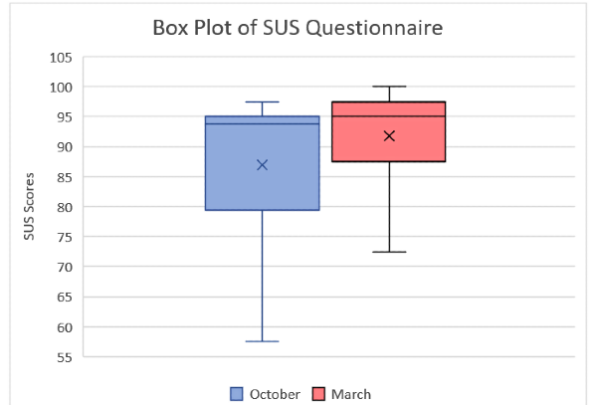
Box plot of SUS scores of the educators from the first (October) and second (March) sessions. SUS: System Usability Scale.

**Figure 13:**
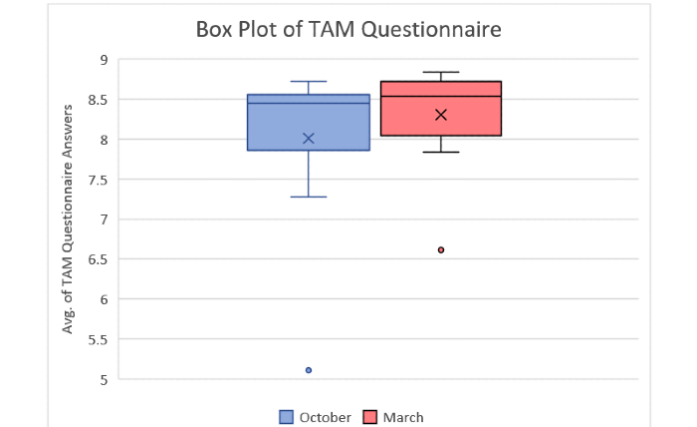
Box plot of TAM questionnaire answers of educators from the first (October) and second (March) sessions. TAM: Technology Acceptance Model.

The TAM questionnaire results of educators from both interviews are displayed in Table S12 in Multimedia Appendix 9. Table S13 in Multimedia Appendix 9 shows the Wilcoxon signed rank test results of educators for both questionnaires. Figures 10 and 11 show the box plots of the false answers of the Memory game and the difference between the correct and false answers, respectively. Figures 12 and 13 show the box plots of the SUS and TAM questionnaires, while Multimedia Appendix 12 and 13 display the individual plots of the SUS and TAM questionnaires.

### The Comments and Suggestions of the Educators

The educators’ feedback (Multimedia Appendix 14) was also analyzed under prototyped game ideas, potential game ideas, and general feedback. For the first segment, educators were asked to comment on current games, game difficulty levels, graphics, and game-related features. Instead of words, one of the educators proposed that game pictures could be used for the first part of the category. Another educator suggested that visuals should enrich the Math game without creating distractions. Other teachers suggested that creating a shape by collecting a gem in the Space game would support children’s sketching ability. She also said that a star or a diamond should be gained instead of points since these symbols correspond to the special education center’s current motivation system.

Educators were also asked if there was any game they want to see in this set of games. One educator suggested a game that enables the players to write letters by tracing a line or an area with fingers. Another educator said that a game based on finding synonyms or antonyms would be helpful to children with dyslexia. Rhythmic counting is indicated as another possible addition to the Math game. A “Spot 7 differences” type of puzzle and jigsaw puzzles were also suggested. Finally, an educator said that a game to improve players’ auditory memory would be beneficial. Three educators said the lack of feedback makes it difficult to feel like they are making progress. Two educators believed that the degrees of difficulty should be varied. Two other educators indicated that holding scores on a leaderboard list and displaying previous scores would be helpful and encouraging to children. One of the educators indicated that a guide for educators or parents would be helpful. Finally, although most educators favored using this method in the classroom, as homework or as auxiliary material, only one educator said she would not use the games until the parents were sufficiently aware of this system and its benefits. Multimedia Appendix 15 shows the most common words or phrases in the comments used by the educators. The most common five words were *feedback*, *audial feedback*, *well-thought*, *visual elements*, and *reward*. The only negative feedback was “Will not use,” which was said due to the educator’s concern about parents’ unfamiliarity with tablet use in special education.

## Discussion

### Principal Findings

In this study, five different serious games and their prototypes were developed to provide training material for children with any type of SpLD. The proposed games were evaluated in a two-phase study consisting of the SUS and TAM questionnaires, and open-ended questions for 23 children with SpLD and 10 educators. The main findings of this study showcase the potentials of using serious games in SpLD training, the impact of adding adaptive difficulty systems that enable personalized training experiences, and the outcomes of player-oriented design where each prototype was structured based on the player statistics and suggestions.

Results on comments and technology acceptance of the students (Table S1 in Multimedia Appendix 9) show that children evaluated the games as easy-to-use and easy-to-learn. *Easy-to-use* was a design criterion, and the results demonstrate that it was accomplished. Since the game was designed with a single control mechanism and a single goal, children did not feel uncomfortable or overwhelmed, even when the game itself was challenging (like the Space game). Children with SpLD aged 7 to 11 years were comfortable with different input types (dragging, tapping, and joystick) when using only one of them during the game. The single goal also mattered because most of these children did not experience more complex games before these interventions (Multimedia Appendix 16). To habituate the children with SpLD to serious games, not only difficulty levels but also the complexity of games were increased in a stepwise fashion.

The results of the questionnaires showed that the outcomes of the feedback-related questions were relatively low. There was no audio feedback in the proposed games, and visual feedback was reduced to prevent any possible distraction. After this result, the final version of the games was modified to have slightly more visual feedback. The questionnaire results of the educators supported these initial findings. Educators remarked that the games were easy to learn and play, but there was a lack of feedback in the final version of the games, although the games included several visual feedbacks. The children’s average points indicated that verbal games (the Word and Category games) had the lowest scores (8.36 and 8.08 out of 10, respectively), which may stem from the duration of the games. Both the Word and Category games took more than four times longer than other games during game play. Since playtime did not include any level mechanism in these games, it became boring for children after a while. In addition, children gave a correct answer in 54.8 seconds (Word game) and 38 seconds (the second part of the Category game), which can be accepted as exceedingly long time durations for students with dyslexia. The point difference between the Category and Word games is based on the visual differences. The Word game has a child avatar and a kitchen illustration, which is a more child-oriented visual theme. The Word game, which only includes a blackboard and some writings, was liked by the children, and they completed the game in their given time period. This result conflicts with Shabbir et al’s [19] rules of serious games for children with dyslexia. Shabbir et al [19] claim that not using a plain background can cause a distraction for children. However, the children’s questionnaires and comments show that game-related static background images or avatars increased the immersion. The kitchen image in the background or the child avatar that asks questions was not mandatory for the Word game, but children stated that they loved both versions. Some of the children suggested a similar visual design for the Category game.

The average points by both participant groups were between 7.9 and 8.84 out of 10. When these results were combined with the questionnaire results, it can be easily said that both participant groups liked all games, and they can accept all parts of the system as a training source. Unlike other studies on serious games for children with SpLD, this study proposes a collection of games to educate children with any type of SpLD. All of the games can be played by children regardless of their SpLD types. A specially designed set of games for these children can provide a complete training source and better experience rather than only playing a game directly related to their disorder or difficulty. The average number of days when students want to play these games was 3.6 per week. This average can be accepted as high since the students’ permission to play games was limited. Almost half of the kids indicated that they were allowed to play games only during the weekend or every other day, and they said that they would play these games in that allocated limited time.

A two-phase SUS and TAM evaluation was provided in this study, although the SUS scores between initial and final versions of the games were demonstrating similar adjective ratings (ie, best imaginable, in our case) as mentioned by Bangor et al [20]. However, it was seen that in the second interview, the average of the SUS was higher, and the SD was lower, which can be considered to demonstrate the condensed results regarding the outcome of the games, where the hesitations and fluctuations from the data were less than the initial versions.

A rule-based adaptive difficulty system was also proposed in serious games that adjust difficulty during the game, in contrast to other studies in the literature that used a system that changes difficulty between two play sessions [19] and used predefined levels, which is limited [21]. The Space game’s adaptive version did not change the results considerably for the students. Moreover, the game’s speed change was practically equal, which means the users nearly had the same statistics at the same difficulty level. It can be interpreted in a couple of ways. The first possible reason for this result can be the adaptive rules or effects not being well-adjusted, which is too difficult to achieve. In that case, the players will usually play in normal difficulty. If adaptive changes affect the game difficulty, too many players will probably increase or decrease their pace to the standard difficulty level. The other possible reason for this result can be the game difficulty being too balanced. The game could have such a difficulty that players can collect the gem and crash into enemies at almost the same rate. However, this is not a desired outcome since children should encounter this situation after a successful game period to feel their progress. The Wilcoxon test for the Memory game results showed that children had better performance in the game’s adaptive version, but it was not statistically significant. Since the adaptive version increases its difficulty while players show good performance, children played a more challenging version and showed better performance, which means that the flow strategy worked. Due to the increased difficulty level, children’s performance could not be statistically different but was still better.

Especially for adaptive games, more tests should be done for different variables. This study includes the modification of only one variable—enemy spawn frequency for the Space game and question screen time for the Memory game—which gives promising results for the future, especially in the Memory game. Children did not realize they were playing faster, and they showed slightly better performance in adaptive versions. More tests can be applied by changing grid numbers or changing the predefined letters in the left panel. During the test of the adaptive games, only 1 student said that he understood the change in the Memory game and explained the correct reason, while others could not explain the change they felt in both games, misjudged them, or did not feel any change at all. Many students showed that the games influenced them by expressing sadness, joy, or surprise during the games—mainly in the Space game. Except for 1 student, the students were eager to replay the games, and they demanded to continue to play after the interventions were complete. In the first interview, 1 student stated that playing the Space game for the second time would be unnecessary, so she did not want to replay the game. The same student did not make such a request in other games or during the second interview. The students wanted different visual themes in the Math game, showing that the game could be diversified visually, not mechanically. The “boss creatures” concept was the most highlighted suggestion of the students, which was proposed as an improvement several times. It may be good to add them as positive feedback in the games that are designed in levels instead of endless games. Besides, although the game ends due to the player’s mistake in endless games, in level-based games, the player’s success leads to a new stage, which changes the feeling at the end of the game. Thus, boss creatures can be good for strengthening the feeling at the end of the episode. Students’ comments on serious games were usually game-related suggestions, and they were not interested in the games’ educational purpose. This is because children did not see these games as educational material, but they accepted these games as standard games, which can be considered in line with the fundamental purpose of the serious game concept.

### Conclusion

This study proposed and tested five serious games, each having a different number of prototypes, specifically developed for children with SpLD. Both educators and students participated in the game-based interventions, and their feedback was recorded. Both participant groups were enthusiastic about the proposed “all-in-one” scheme. Besides, different design requirements that have been listed in previous studies were investigated with an additional adaptive difficulty level. These adjustments and personalized approaches increased children with dyslexia’s immersion in the games. One of the study’s contributions was the use of serious games to target various SpLD types. Each child participant had more than one form of SpLD, and playing games that focus on dyslexia or dyscalculia has been beneficial to them instead of playing games that focus on only one of these difficulties.

Furthermore, playing games like the Memory and Space games and education-oriented question-and-answer games resulted in increased positive feedback and excitement. Furthermore, the students mentioned that they preferred to play in a general system rather than a system focusing only on an area where they already had difficulties. The teachers’ overall impressions were highly positive, and most of them intend to use these games as part of their curriculum’s supplementary material. This integration will enable the games to be tested for a longer period so that new enhancements can be added to the proposed games with additional evaluations to measure their educational impact.

## Appendix

Multimedia Appendix 1 The workflow diagram of this study.

Multimedia Appendix 2 Screenshot of the picture version of the Memory game from Prototype IV.

Multimedia Appendix 3 Screenshot of the first part of the Category game from Prototype II.

Multimedia Appendix 4 Screenshot of the second part of the Category game from Prototype II.

Multimedia Appendix 5 Screenshot of the second part of the Category game with an alternative keyboard layout from Prototype III for the theme “Clothes".

Multimedia Appendix 6 The optimal game play corridor and the golden path figure are adapted from Thomas 2010.

Multimedia Appendix 7 Visual diagram of the adaptive difficulty system.

Multimedia Appendix 8 Screenshot of the letter version of the Memory game from Prototype III.

Multimedia Appendix 9 Supplementary files and tables on game statistics and results of the questionnaires.

Multimedia Appendix 10 Student feedback illustration map.

Multimedia Appendix 11 Student feedback word cloud.

Multimedia Appendix 12 Individual plot of System Usability Scale scores of educators from the first (October) and second (March) sessions.

Multimedia Appendix 13 Individual plot of Technology Acceptance Model questionnaire answers of educators from the first (October) and second (March) sessions.

Multimedia Appendix 14 Educator feedback illustration map.

Multimedia Appendix 15 Educator feedback word cloud.

Multimedia Appendix 16 Photo of a child during the intervention.

## Abbreviations

IDEA: Individuals with Disabilities Education Act
LD: learning disorder
SpLD: specific learning difficulties
SUS: System Usability Scale
TAM: Technology Acceptance Model
