# Supplementary figures and images for: Developing Adaptive Serious Games for Children With Specific Learning Difficulties: A Two-phase Usability and Technology Acceptance Study
Source: JMIR Serious Games. 2021 May 31;9(2):e25997. doi: 10.2196/25997 (PMC8204245; doi:10.2196/25997)

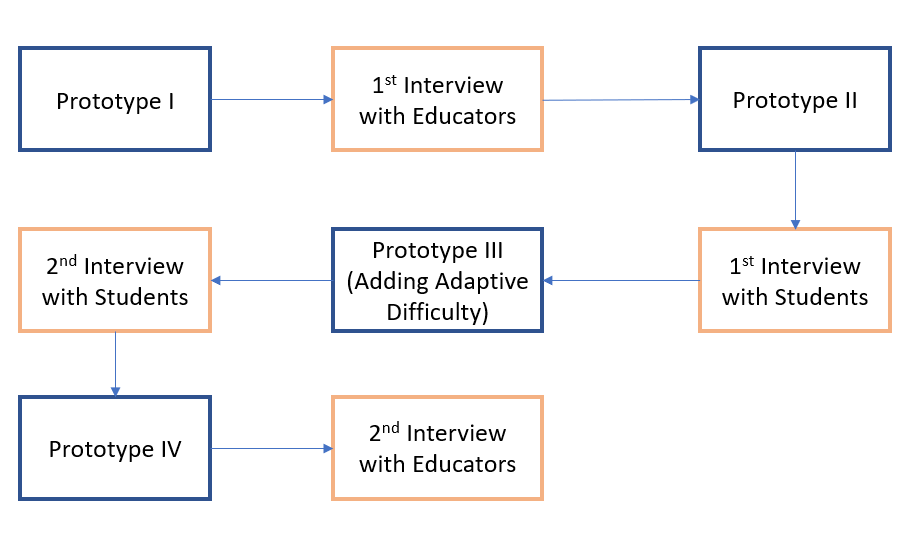

Supplement: Multimedia Appendix 1 [file games_v9i2e25997_app1.png]

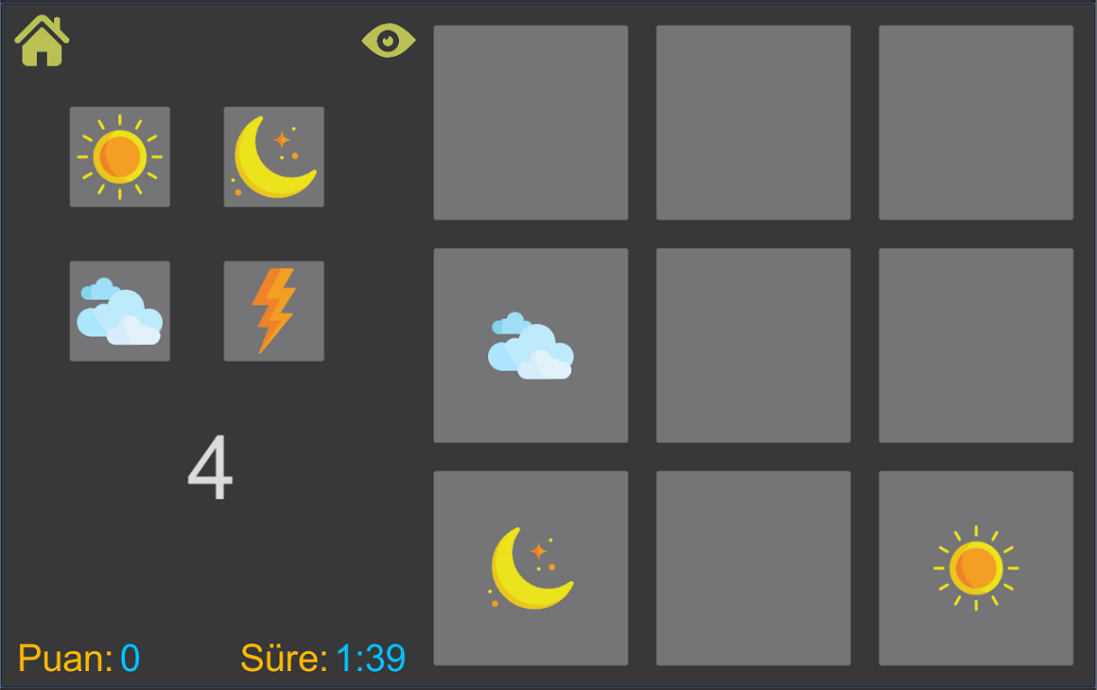

Supplement: Multimedia Appendix 2 [file games_v9i2e25997_app2.png]

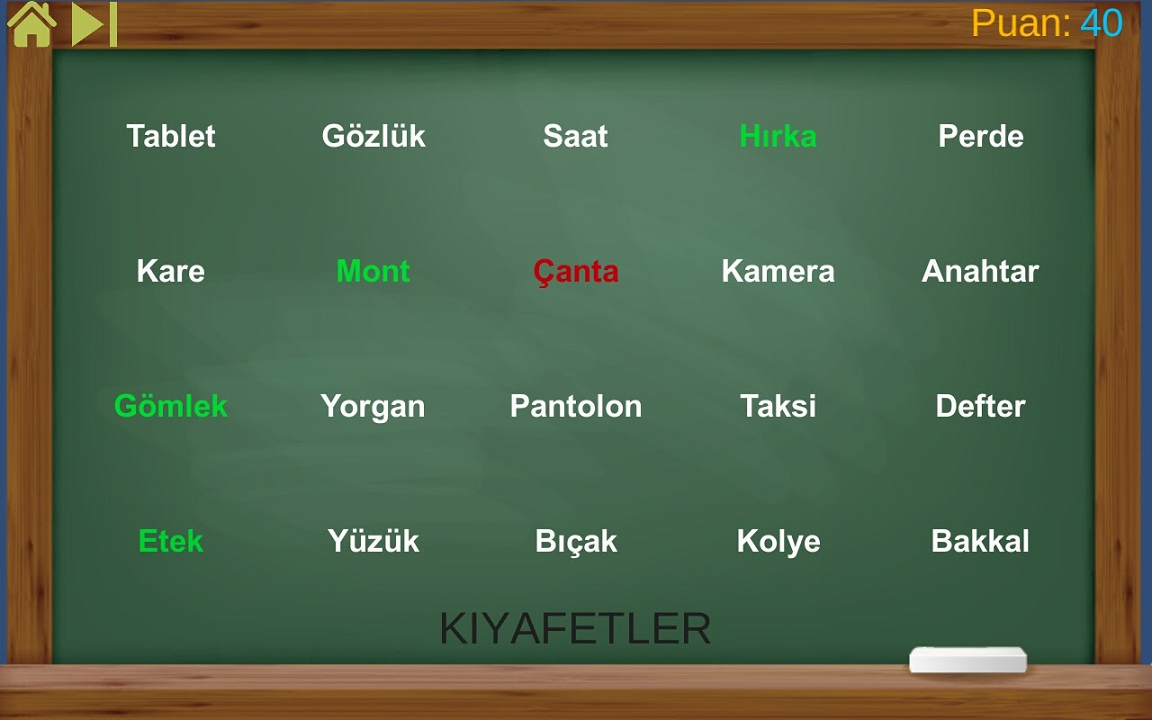

Supplement: Multimedia Appendix 3 [file games_v9i2e25997_app3.png]

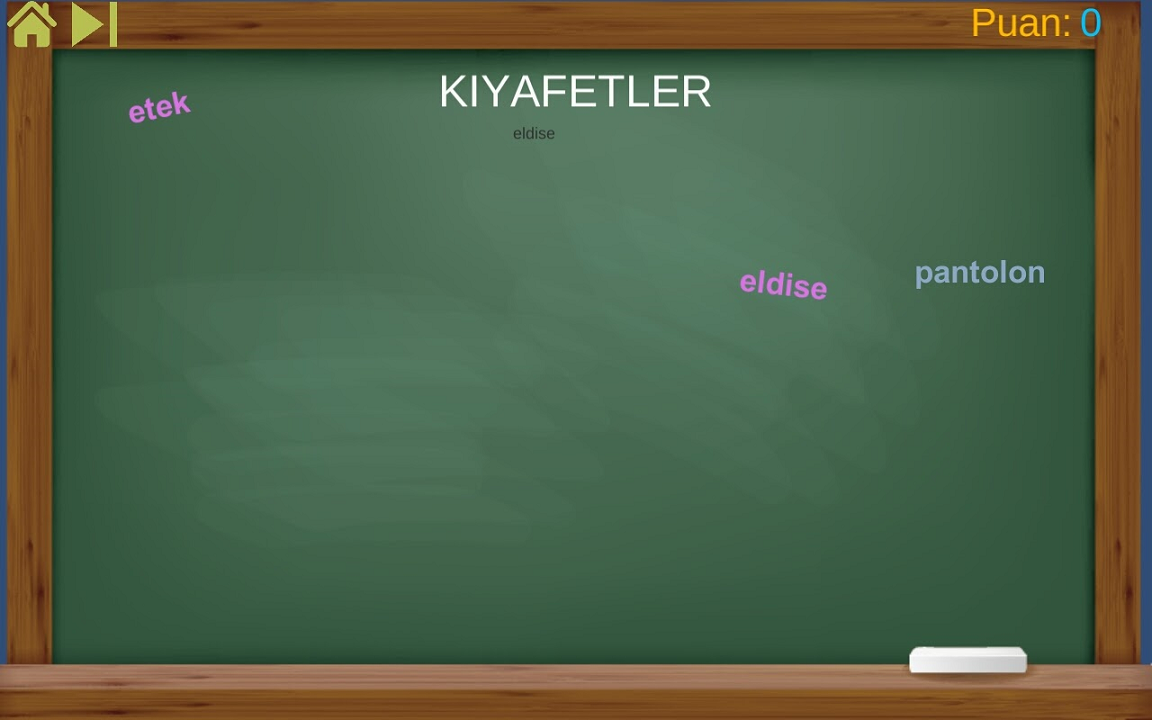

Supplement: Multimedia Appendix 4 [file games_v9i2e25997_app4.png]

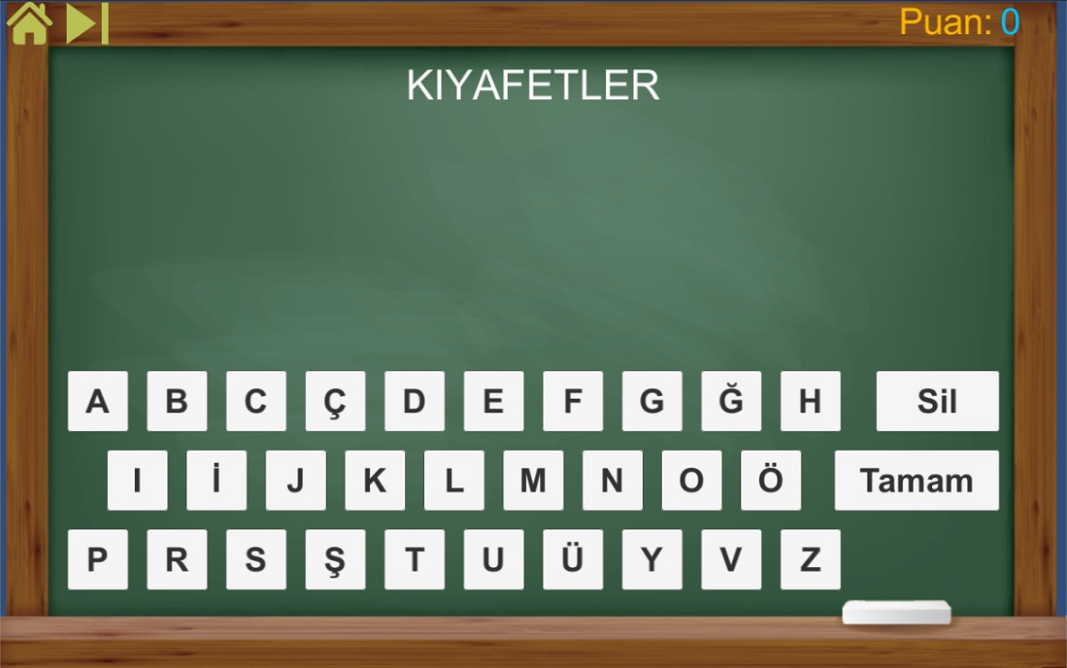

Supplement: Multimedia Appendix 5 [file games_v9i2e25997_app5.png]

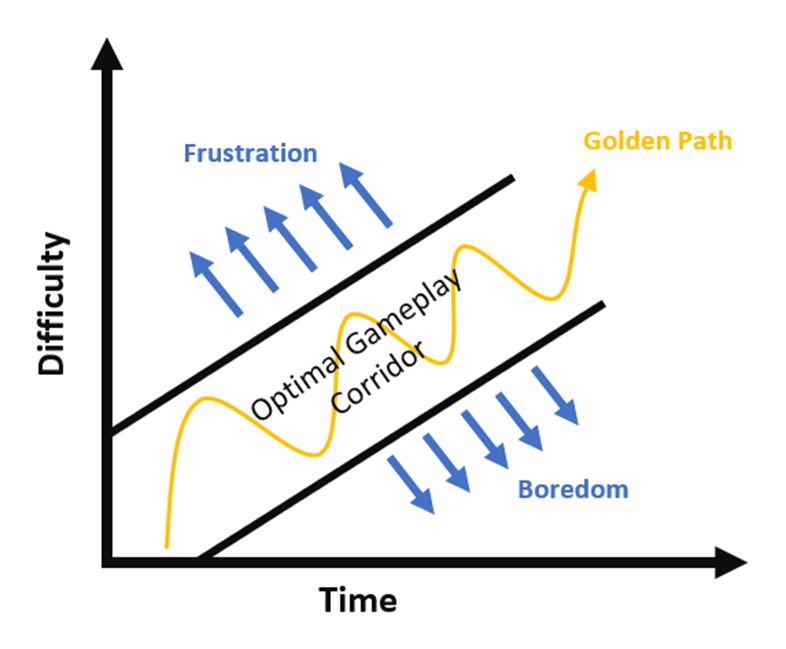

Supplement: Multimedia Appendix 6 [file games_v9i2e25997_app6.png]

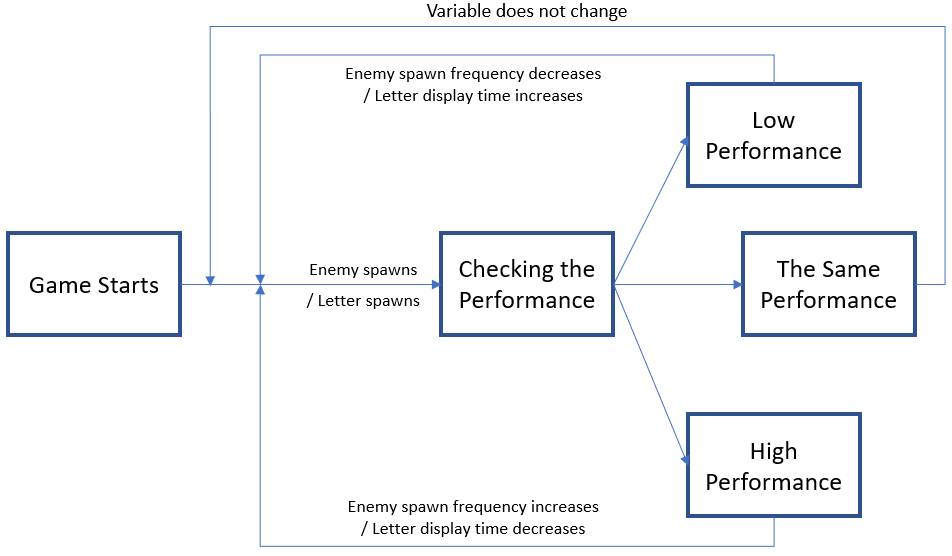

Supplement: Multimedia Appendix 7 [file games_v9i2e25997_app7.png]

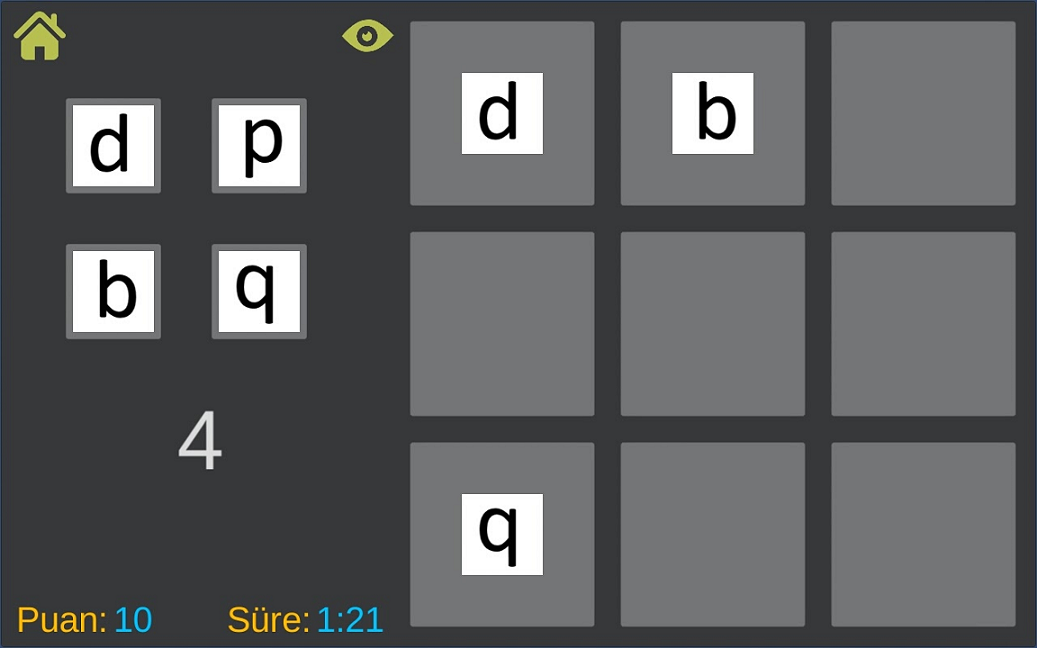

Supplement: Multimedia Appendix 8 [file games_v9i2e25997_app8.png]

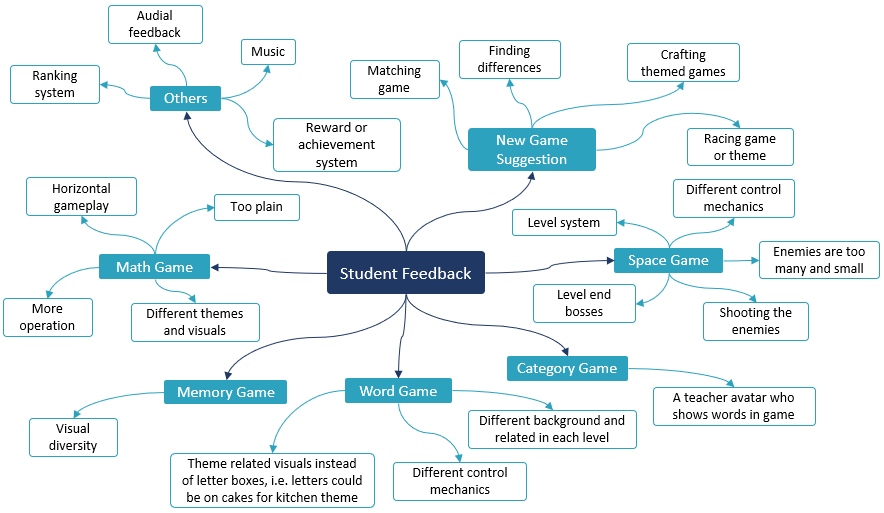

Supplement: Multimedia Appendix 10 [file games_v9i2e25997_app10.png]

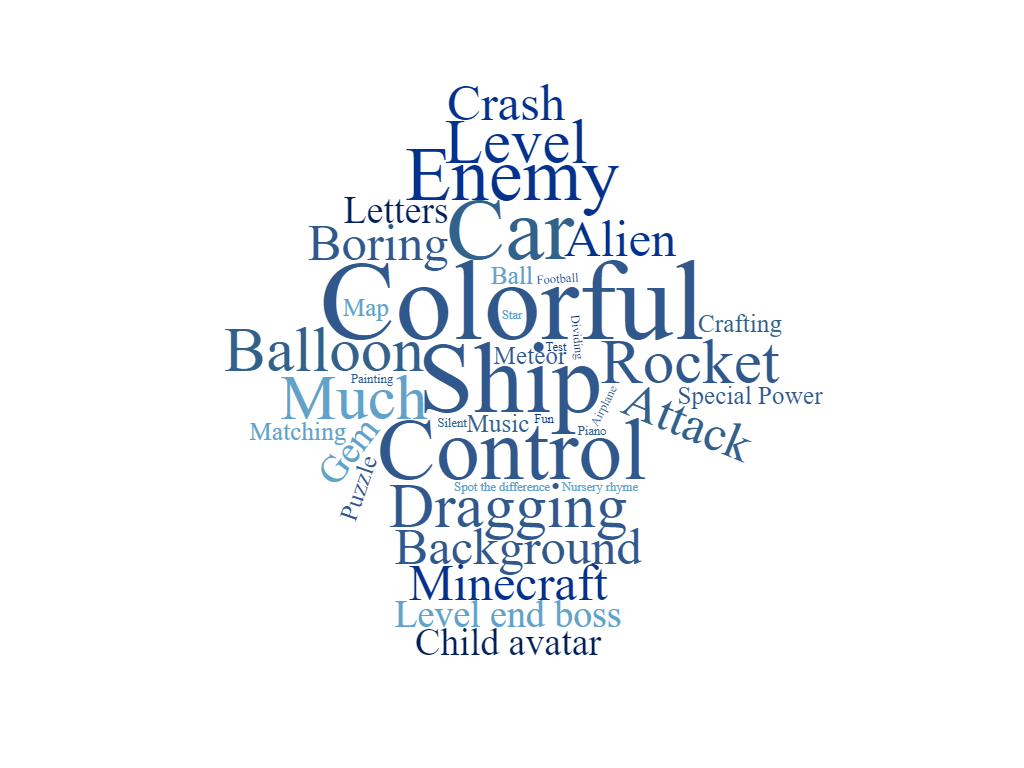

Supplement: Multimedia Appendix 11 [file games_v9i2e25997_app11.png]

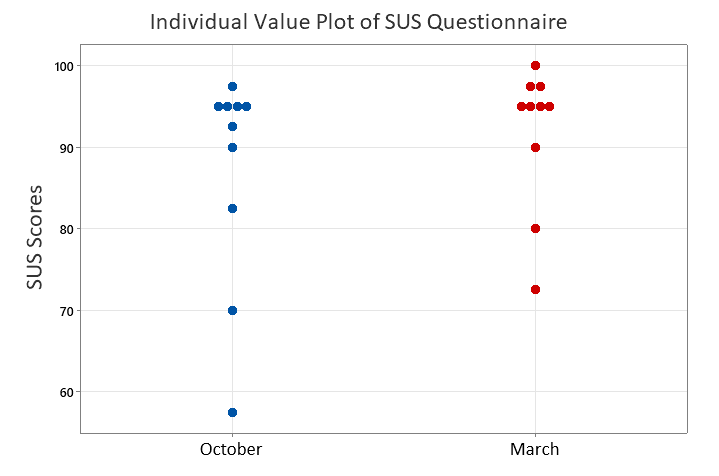

Supplement: Multimedia Appendix 12 [file games_v9i2e25997_app12.png]

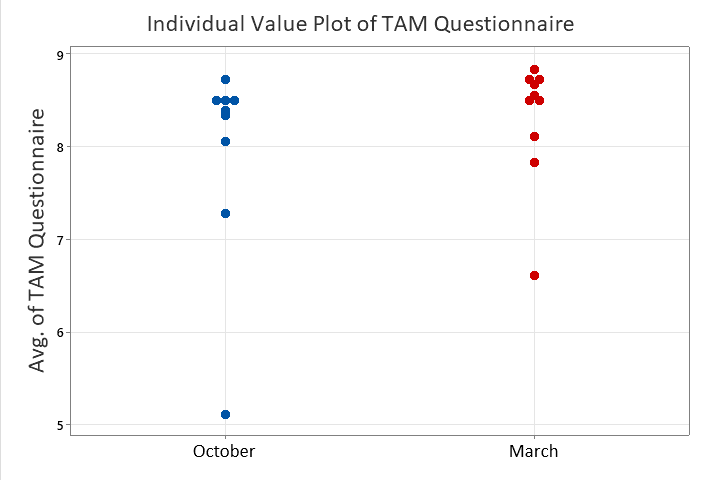

Supplement: Multimedia Appendix 13 [file games_v9i2e25997_app13.png]

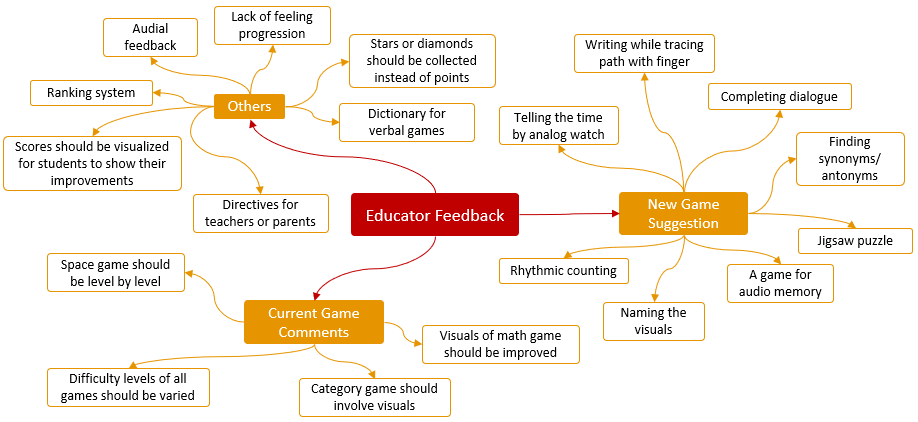

Supplement: Multimedia Appendix 14 [file games_v9i2e25997_app14.png]

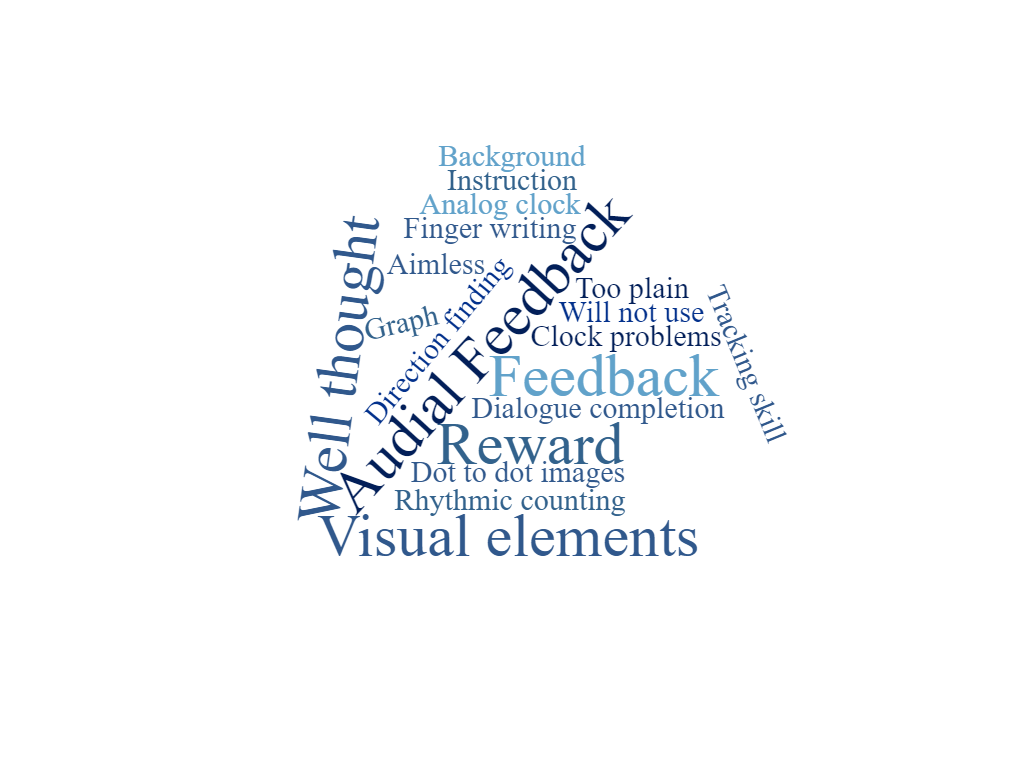

Supplement: Multimedia Appendix 15 [file games_v9i2e25997_app15.png]

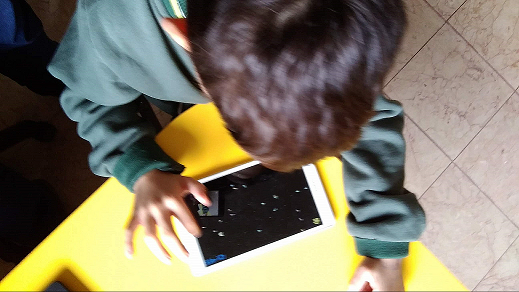

Supplement: Multimedia Appendix 16 [file games_v9i2e25997_app16.png]
