# Supplementary material for: Developing Adaptive Serious Games for Children With Specific Learning Difficulties: A Two-phase Usability and Technology Acceptance Study
Source: JMIR Serious Games. 2021 May 31;9(2):e25997. doi: 10.2196/25997 (PMC8204245; doi:10.2196/25997)
Supplement: Multimedia Appendix 9 [file games_v9i2e25997_app9.docx]

**Multimedia Appendix 9.** Supplementary files and tables on game statistics and results of the questionnaires.

**Table S1:** The results of the TAM Questionnaire for students. Participants were also asked to give ratings to each game on a 0 to 10 scale. The games’ ratings are on a 0 to 10 scale; a higher score indicates a more liked game.

| Technology Acceptance Model - First Interview | |
| --- | --- |
| Positive Statements | 9.07 ± 0.56 |
| Game Scores - First Interview | |
| Math Game | 8.52 ± 2.26 |
| Word Game | 8.36 ± 2.46 |
| Memory Game | 8.44 ± 2.32 |
| Space Game | 8.84 ± 2.29 |
| Category Game | 8.08 ± 2.92 |

The playtime of the games was recorded, and the average playing times are shown in Table 2.

**Table S2:** Average Playing Time of Games - Average and standard deviation of playing time in the first interview with students.

|  | Average Play Time in a Session (seconds) |
| --- | --- |
| Math Game | 99.57 ± 2.00 |
| Word Game | 543.27 ± 210.81 |
| Memory Game | 725.63 ± 273.09 |
| Space Game | 93.94 ± 24.61 |
| Category Game | 58.35 ± 9.10 |

**Table S3:** Memory Game Statistics - Mean and standard deviation results of data of the first session with students according to how many times they played in that session.

| Order of Play Sessions | Number of Correct Answers | Number of False Grids | Number of False Color/Arrow Markings |
| --- | --- | --- | --- |
| First Time | 4.88 ± 1.24 | 0.16 ± 0.46 | 0.88 ± 1.37 |
| Second Time | 4.64 ± 0.93 | 0.24 ± 0.51 | 1.92 ± 3.49 |
| Third Time | 3.91 ± 1.10 | 0.74 ± 1.59 | 2.26 ± 2.23 |

**Table S4:** Memory Game Statistics - Mean and standard deviation results of the first session’s data with students according to difficulty and version.

| Version - Difficulty | Number of Students | Number of Correct Answers | Number of False Grids | Number of False Color/Arrow Markings |
| --- | --- | --- | --- | --- |
| Color - 1 Grid | 20 | 5.10 ± 1.26 | 0.20 ± 0.51 | 0.85 ± 1.42 |
| Color - 2 Grids | 13 | 4.15 ± 0.53 | 0.23 ± 0.42 | 0.77 ± 0.80 |
| Color - 3 Grids | 17 | 3.76 ± 1.11 | 0.88 ± 1.78 | 2.12 ± 2.49 |
| Arrow - 1 Grid | 3 | 5.67 ± 0.47 | 0.00 ± 0.00 | 0.00 ± 0.00 |
| Arrow - 2 Grids | 16 | 4.69 ± 0.98 | 0.19 ± 0.53 | 3.25 ± 3.98 |
| Arrow - 3 Grids | 5 | 4.20 ± 0.98 | 0.40 ± 0.80 | 1.40 ± 1.36 |

**Table S5:** Space Game Statistics - Mean and standard deviation results of data of the first session with students according to how many times they played in that session.

| Order of Play Sessions | Number of Collected Gems | Number of Crashed Enemies | Average Play Time (sec) |
| --- | --- | --- | --- |
| First Time | 7.36 ± 5.07 | 11.36 ± 2.68 | 88.44 ± 23.22 |
| Second Time | 11.58 ± 6.16 | 13.46 ± 4.82 | 99.67 ± 24.71 |

**Table S6:** Math Game Statistics - Mean and standard deviation results of the first session’s data with students according to how many times they played in that session.

| Order of Play Sessions | Number of Correct Answers | Number of False Answers | Number of Unanswered Questions | Number of Total Answers |
| --- | --- | --- | --- | --- |
| First Time (Summation Only) | 11.75 ± 4.15 | 1.46 ± 2.68 | 0.25 ± 0.52 | 13.46 ± 3.62 |
| Second Time (Summation and Subtraction | 10.46 ± 3.07 | 2.46 ± 3.21 | 0.29 ± 0.61 | 13.21 ± 3.98 |

**Table S7:** Word Game Statistics - Mean and standard deviation results of the first session’s data with students in 60 seconds and per 1 correct.

|  | Number of Correct Answers out of 10 | Number of False Answers out of 10 | Number of Unanswered Questions out of 10 | Total Time (in seconds) |
| --- | --- | --- | --- | --- |
| Per 60 Seconds | 1.09 ± 0.48 | 0.61 ± 0.60 | 0.01 ± 0.04 | 60.00 ± 0.00 |
| Per 1 Correct | 1.00 ± 0.00 | 0.56 ± 0.49 | 0.01 ± 0.05 | 54.80 ± 21.49 |

**Table S8:** Category Game (Part 1) Statistics - Mean and standard deviation results of the first session’s data with students per 60 seconds and per 1 correct.

|  | Number of Correct Answers out of 25 | Number of False Answers out of 25 | Total Time (in seconds) |
| --- | --- | --- | --- |
| Per 60 Seconds | 4.98 ± 3.17 | 1.01 ± 0.90 | 60.00 ± 0.00 |
| Per 1 Correct | 1.00 ± 0.00 | 0.20 ± 0.23 | 12.05 ± 4.74 |

**Table S9:** Category Game (Part 2) Statistics - Mean and standard deviation results of the first session’s data with students in 60 seconds and per 1 correct.

|  | Number of Correct Answers | Number of False Answers | Number of Typos | Number of Total Answers | Total Time (sec) |
| --- | --- | --- | --- | --- | --- |
| Per 60 Seconds | 1.58 ± 0.79 | 0.03 ± 0.06 | 0.15 ± 0.15 | 1.76 ± 0.78 | 60.00 ± 0.00 |
| Per 1 Correct | 1.00 ± 0.00 | 0.02 ± 0.04 | 0.10 ± 0.10 | 1.13 ± 0.11 | 38.00 ± 15.09 |

**Table S10:** Memory Game Statistics - Mean and standard deviation results of the second session data with students.

|  | Number of Correct Answers (per 10 seconds) | Number of False Grids (per 10 seconds) | Number of False Letters (per 10 seconds) |
| --- | --- | --- | --- |
| Normal | 0.70 ± 0.16 | 0.08 ± 0.11 | 0.51 ± 0.45 |
| Adaptive | 0.72 ± 0.18 | 0.07 ± 0.08 | 0.45 ± 0.34 |

**Table S11:** Space Game Statistics - Mean and standard deviation results of the second session data with students.

|  | Collected Gems (per 10 seconds) | Crashed Enemies (per 10 seconds) | Average Play Time (sec) |
| --- | --- | --- | --- |
| Normal | 1.20 ± 0.29 | 1.32 ± 0.44 | 103.92 ± 20.47 |
| Adaptive | 1.09 ± 0.24 | 1.35 ± 0.32 | 94.36 ± 17.63 |

**Table S12:** Technology Acceptance Model Questionnaire - Mean and standard deviation results of both interviews with educators. The questionnaire answers are on a 1 to 10 scale; a higher score indicates a stronger agreement on statements. Game Ratings - Mean and standard deviation results of both interviews with educators. The games’ ratings are on a 0 to 10 scale; a higher score indicates a more liked game.

| Technology Acceptance Model Results of the Educators | | |
| --- | --- | --- |
|  | First Interview | Second Interview |
| Positive Statements | 8.41 ± 0.87 | 8.72 ± 0.64 |
| Game Scores of the Educators | | |
|  | First Interview | Second Interview |
|  |  |  |
| Math Game | 8.00 ± 2.19 | 8.00 ± 2.19 |
| Word Game | 8.50 ± 1.57 | 8.60 ± 1.43 |
| Memory Game | 7.80 ± 2.93 | 8.50 ± 2.06 |
| Space Game | 7.90 ± 1.51 | 8.80 ± 1.40 |
| Category Game | 7.80 ± 2.09 | 7.90 ± 1.97 |

**Table S13:** Wilcoxon Signed Ranks Test - Results of test between the educators’ interviews on both questionnaires.

|  | Z | Asymp. Sig. (two-tailed) |
| --- | --- | --- |
| SUS Scores | -2.060 | 0.039 |
| TAM Answers | -2.310 | 0.021 |
